# *De novo* Transcriptome Assembly, Gene Annotation and SSR Marker Development in the Moon Seed Genus *Menispermum* (Menispermaceae)

**DOI:** 10.3389/fgene.2020.00380

**Published:** 2020-05-08

**Authors:** Faiza Hina, Gulbar Yisilam, Shenyi Wang, Pan Li, Chengxin Fu

**Affiliations:** ^1^Laboratory of Systematic and Evolutionary Botany and Biodiversity, College of Life Sciences, Zhejiang University, Hangzhou, China; ^2^Department of Botany, University of Wisconsin–Madison, Madison, WI, United States

**Keywords:** *Menispermum*, Illumina high-seq transcriptome, *de novo* assembly EST-SSR, *Menispermum canadense*, *Menispermum dauricum*, population genetic diversity

## Abstract

The moonseed genus *Menispermum* L. (Menispermaceae) is disjunctly distributed in East Asia and eastern North America. Although *Menispermum* has important medicinal value, genetic and genomic information is scarce, with very few available molecular markers. In the current study, we used Illumina transcriptome sequencing and *de novo* assembly of the two *Menispermum* species to obtain in-depth genetic knowledge. From *de novo* assembly, 53,712 and 78,921 unigenes were generated for *M. canadense* and *M. dauricum*, with 37,527 (69.87%) and 55,211 (69.96%) showing significant similarities against the six functional databases, respectively. Moreover, 521 polymorphic EST-SSRs were identified. Of them, 23 polymorphic EST-SSR markers were selected to investigate the population genetic diversity within the genus. The newly developed EST-SSR markers also revealed high transferability among the three examined Menispermaceae species. Overall, we provide the very first transcriptomic analyses of this important medicinal genus. In addition, the novel microsatellite markers developed here will aid future studies on the population genetics and phylogeographic patterns of *Menispermum* at the intercontinental geographical scale.

## Introduction

*Menispermum* L. (moonseed) is a small genus (∼2 spp.) of deciduous climbing woody lianas in the family Menispermaceae. The genus exhibits a classic East Asian-Eastern North American disjunct pattern, with *M. dauricum* DC. native to East Asia and *M. canadense* L. occurring in eastern North America (Xiang et al., 2000; Ortiz et al., 2016). Both species are a typical element of temperate forests in East Asia/eastern North America, where they are widespread and abundant (Londre and Schnitzer, 2006).

*Menispermum canadense* (Canadian moonseed) is widely distributed in the lowland forests of eastern North America, west to Manitoba and Oklahoma (Purrington and Horn, 1993), while *M. dauricum* (Siberian moonseed) is widespread from eastern China to Siberia, Korea, and Japan. Rhizomes of *M. dauricum* are used as a crude drug in Traditional Chinese Medicine (TCM) known as “Bei-Dou-Gen” or “Bian-Fu-Ge-Gen” in China, which is useful for treating tonsillitis, rheumatic arthralgia, diarrhea, dysentery, gastroenteritis, cardiovascular, and thrombosis disorders. More than 25 phenolic alkaloids have been isolated from *Menispermum*, of which several are common in both species. Phenolic alkaloids from *M. dauricum* had broad pharmacological effects, for instance, anti-inflammation, anti-thrombosis, antioxidant, analgesia, treating high blood pressure and cardiovascular disorder, anti-arrhythmic, shielding neurons of cerebral ischemia, anti-tumor, and improving learning disabilities (Su et al., 2004, 2007; Guo et al., 2015). However, we still lack overall genetic knowledge on *Menispermum*, despite its medicinal importance.

Microsatellites (or simple sequence repeats, SSRs) are characterized by ample distribution in the genome, high polymorphism, co-dominance, reproducibility, and testable neutrality, and have thus been commonly used for assessing the genetic similarity among individuals or closely related taxa (Estoup et al., 1999; Guichoux et al., 2011). Microsatellites can be classified into genomic SSRs (gSSR) and expressed sequence tag SSRs (EST-SSR), depending on whether they are recovered from random genomic sequences or transcribed RNA sequences (Song et al., 2012). EST-SSRs are derived from the most conserved regions of the genome and thus have a higher transferability rate across the related species than that of gSSRs. Microsatellites have been extensively used in genomics, genetic diversity evolution, phylogenetics, comparative genetic mapping, and molecular breeding (Naghavi et al., 2007). So far, no microsatellite markers have been developed for either *M. canadense* or *M. dauricum*.

Transcriptome, or RNA sequencing (RNA-seq), technology is an efficient molecular technique for studying the evolution of species, determining differentially expressed genes, exploring the population dynamics, and comparative genetic mapping (Guo et al., 2015; Rai et al., 2016; Arora and Narula, 2017). *De novo* transcriptome assembly has become a revolutionary mode for high-throughput sequencing in life sciences research (Mardis, 2008; Davey et al., 2011). Transcriptome sequencing not only allows quick and comprehensive analyses of the plant genome but also presents an easy and effective way of developing a large number of EST-SSR markers (Lister et al., 2009; Tan et al., 2013).

To further understand the genetic and genomic background of *Menispermum*, we sequenced the transcriptomes of both *M. canadense* and *M. dauricum*, and we aim to (i) characterize their transcriptomes, (ii) develop polymorphic EST-SSRs in *Menispermum*, and (iii) test their transferability in the family Menispermaceae. The transcriptomic analyses presented here will offer foundational genetic information for future research, utilization, and protection of *Menispermum*.

## Results

### Transcriptome Sequencing and *de novo* Assembly

**FIGURE 1:**
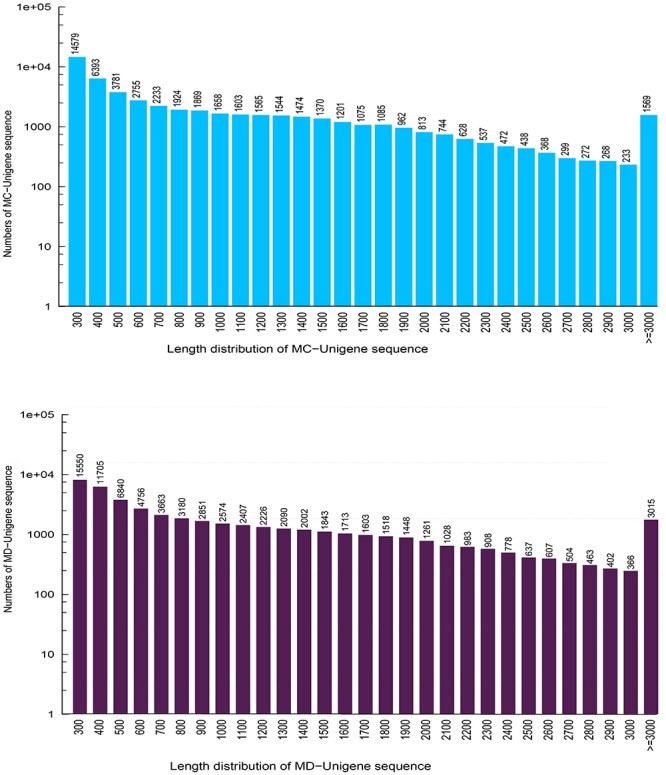
Length distribution of all unigenes in *Menispermum canadense* (MC) and *M. dauricum* (MD). The x-axis represents the lengths of all the unigenes, and the y-axis represents the numbers of unigenes with certain length.

In this research, about 53.79 and 57.15 Mb raw reads were generated for *M. canadense* and *M. dauricum*, respectively. The raw reads were deposited in the NCBI (National Centre for Biotechnology Information) SRA (Sequence Read Archive) database (*M. canadense* SRP166813 and *M. dauricum*: SRP166814). Through rigorous quality checking and data filtering, 44.60 and 45.22 Mb high-quality clean reads were left, respectively. Through *de novo* assembly, 86,883 and 104,064 transcripts were obtained with a mean length of 799 and 945 bp in *M. canadense* and *M. dauricum*, respectively (Table 1). The transcripts (Supplementary Figure S1) were then clustered into unigenes with total lengths ranging from 300 to >3,000 bp (unigenes <300 bp length were discarded) in both *M. canadense* and *M. dauricum*. In *M. canadense*, of all 53,712 unigenes, 14,579 were of 300 bp, while 1,569 were longer than 3,000 bp (Figure 1). From 78,921 unigenes of *M. dauricum*, 15,550 had 300 bp length, and only 3,015 were longer than 3,000 bp. Unigenes present an ample source of information for the identification of genes and molecular markers.

**TABLE 1:** Summary of *de novo* transcriptome assembly of *Menispermum canadense* and *M. dauricum*.

**Categories**	**Features**	***M. canadense***	***M. dauricum***
Raw reads	Total raw reads (M)	57.15	53.79
	Total clean reads (M)	44.60	45.22
	Total Clean bases (Gb)	6.69	6.78
	Clean reads Q20 (%)	97.09	98.09
	Clean reads Q30 (%)	92.15	94.83
Transcripts	Total number of transcripts	86,883	104,064
	Total length of transcripts (bp)	69,428,938	98,356,172
	Mean length of transcripts (bp)	799	945
	N50 value of transcripts	1,431	1,528
	GC (%)	42.59	46.36
Unigenes	Total number of unigenes	53,712	78,921
	Total length of unigenes (bp)	49,461,581	77,153,863
	Mean length unigenes (bp)	920	978
	N50 value of unigenes	1,519	1,583
	GC (%)	42.50	45.18

### Functional Annotation of Unigenes

*De novo* assembled unigenes of *M. canadense* and *M. dauricum* were functionally annotated against the six functional public databases, Nt, Nr, COG, GO, KEGG, and Swiss–Prot. All nucleotide sequences were obtained by splicing, and the BLAST algorithm (*E* < 1E-5) was used for comparison and to get a similar sequence and corresponding annotation.

**FIGURE 2:**
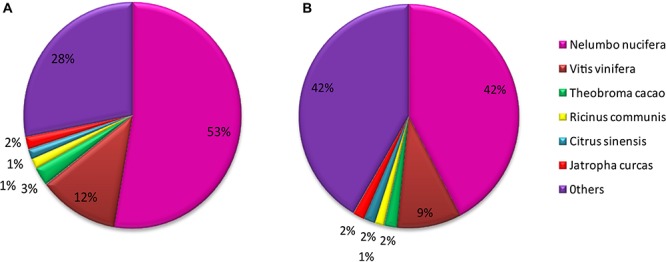
Characteristics of homology analysis in *Menispermum* unigenes against the non-redundant protein database with an E-value of 10^–5^. **(A)** Species based distribution of the top BLASTx hits for 548 each assembled unigenes in *M. canadense*. **(B)** Species based distribution of the top BLASTx hits for each assembled unigenes in *M. dauricum*.

Of *M. canadense* and *M. dauricum* unigenes, 35,842 (66.73%) and 52,180 (66.11%) show significant homology with the proteins in the Nr database, while 24,302 (45.25%) and 40,554 (51.38%) of the control sequences match with the entries in the Nt database (Table 2), respectively. As for the species distribution of Nr annotations (Figure 2A) of *M. canadense* and *M. dauricum* (Figure 2B), *Nelumbo nucifera* (Nelumbonaceae) has the highest similarity score (53.49 and 42.3%, respectively), followed by *Vitis vinifera* (Vitaceae; 12.01 and 9.4%) and *Theobroma cacao* (Malvaceae; 2.7 and 1.9%).

**TABLE 2:** Unigenes functional annotation summary of *Menispermum canadense* and *M. dauricum*, against 6 functional databases.

**Values**	**Total**	**Nr**	**Nt**	**Swiss-Prot**	**KEGG**	**KOG**	**GO**	**Overall**
***M. canadense***								
Number	53,712	35,842	24,302	23,262	26,198	27,264	17,110	37,527
Percentage	100	66.73	45.25	43.31	48.77	50.76	31.86	69.87
***M. dauricum***								
Number	78,921	52,180	40,554	37,988	33,065	24,402	33,051	55,211
Percentage	100	66.12	51.38	48.13	41.90	30.92	41.88	69.96

**FIGURE 3:**
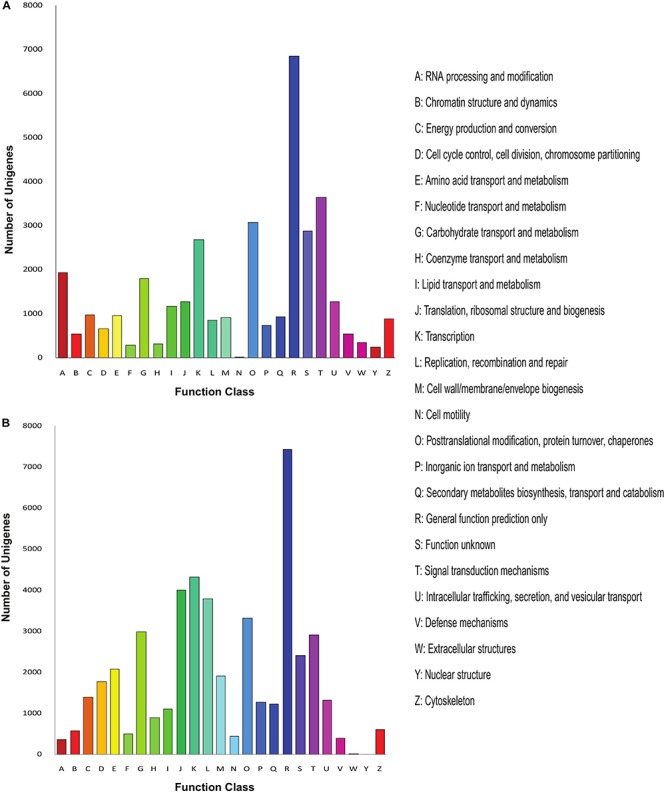
COG analysis of the unigene sequences of *Menispermum*. **(A)** COG analysis of the unigene sequences of *M. canadense*. **(B)** COG analysis of the unigene sequences of *M. dauricum*. The x-axis indicates the function class while y-axis indicates the number of unigenes in specific functional group.

Further, we annotated the unigenes of both *Menispermum* species with the COG database and calculated the unigene distribution based on the 25 functional groups, including cellular structure, metabolic functions, signal transduction, etc. In *M. canadense*, general function prediction (6,861, 19%) represents the largest group, followed by signal transduction mechanism with 3,649 genes (10%, Figure 3A), while in *M. dauricum*, general function prediction, with 7,432 genes (16%), is again the largest group, followed by transcription (4,333 genes, 9%), and translation, ribosomal structure, and biogenesis (4,011 genes, 8%). Cell motility is the smallest group in *M. canadense*, while in contrast in *M. dauricum*, nuclear structures and extracellular structures are classified as the smallest groups.

**FIGURE 4:**
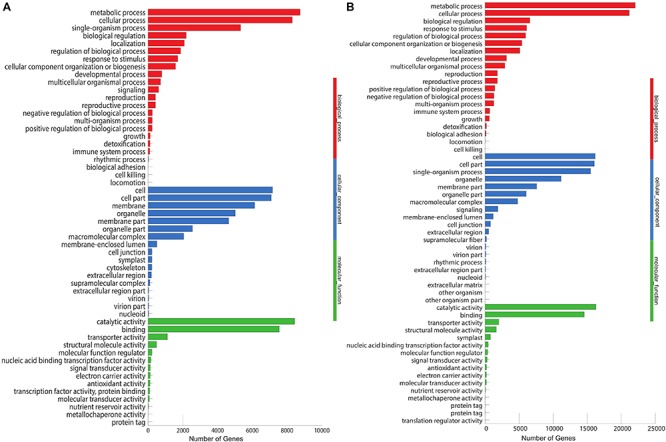
Gene Ontology (GO classification of assembled unigenes of *Menispermum*. **(A)** A summary of GO analysis of the *M. canadense*. **(B)** Summary of GO analysis of the *M. dauricum*. The y-axis indicates genes in the specific categories of the three main categories while x-axis indicates the number of genes in the category.

The unigenes aligned to the Nr database were further annotated to the GO database. We annotated 17,110 (31.86%) unigenes into three major categories: biological process (7,167, 28%), cellular component (8,436, 33%), and molecular functions (10,056, 39%), with 54 subcategories (Figure 4A). Most of the unigenes in the biological processes are specified for the metabolic process (8,748) and cellular processes (8,303), while catalytic activity (8,456) and binding (7,550) are the major subcategories in the metabolic functions. In *M. dauricum*, 33,051 unigenes were assigned to GO functional groups (Figure 4B). The distribution of ontology categories is consistent with most of the sequenced plant transcriptomes, such as *Vaccinium cyanococcus* (Rowland et al., 2012) and *Salix integra* (Shi et al., 2016). Mostly sequenced unigenes are accountable for the fundamental biological metabolism processes, cell, and cell parts demonstrated from the GO annotation for *Menispermum*.

**FIGURE 5:**
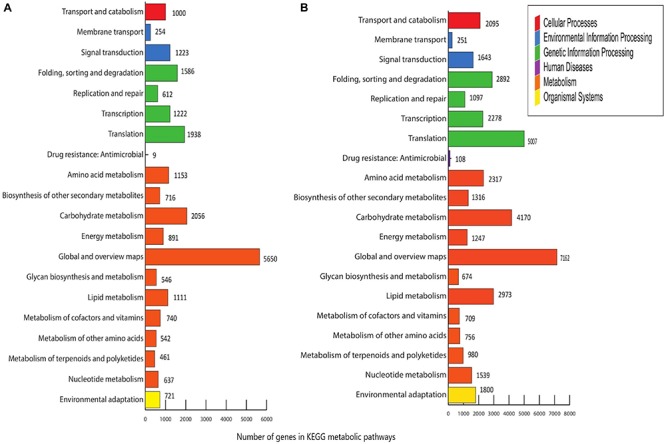
KEGG metabolic pathways of *Menispermum*. **(A)** Metabolic pathways of assembled unigenes of *M. canadense*. **(B)** Metabolic pathways of assembled unigenes of *M. dauricum*. The y-axis is the name of metabolic pathway, and the x-axis is the ratio of the number of the genes.

*Menispermum canadense* and *M. dauricum* transcriptomes were also analyzed against the KEGG database, with 26,198 (48.77%) and 33,065 (41.09%) unigenes identified, respectively. Unigenes are significantly assigned to 135 metabolic pathways. In *M. canadense*, metabolic pathways are categorized into six main divisions (Figure 5A), of which metabolisms is the largest division (with 14,503 genes), followed by genetic information processing (5,450) and environmental information processing (1,477), cellular processing (1,000), organismal systems (721), and human diseases (92) pathways. *M. dauricum* also shows the same divisions, with 25,643 genes in the largest category, metabolisms, followed by genetic information process with 11,382 genes (Figure 5B). In addition, 23,262 (43.31%) and 37,988 (48.13%) unigenes match with the Swiss-Prot database in *M. canadense* and *M. dauricum*, respectively. These functional annotations present valuable information for understanding gene structures and functions and developmental and biochemical pathways in *Menispermum*.

### Identification of Candidate Polymorphic EST-SSR

**FIGURE 6:**
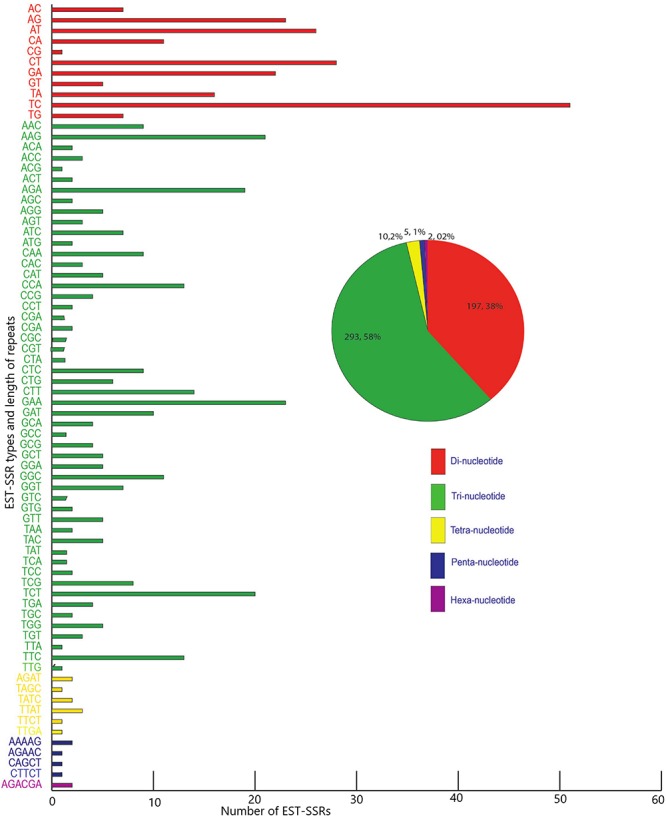
Frequency and distribution of candidate polymorphic EST-SSR from the transcriptome of *Menispermum canadense* and *M. dauricum*.

Five hundred and twenty-one candidate polymorphic EST-SSRs were successfully mined out from the transcriptomes of *M. canadense* and *M. dauricum*, with a mean length of 20 bp (Supplementary Table S1). Of these EST-SSRs, tri-nucleotide repeats (TNR) are the most abundant repeat type (307; 59.38%), followed by di- (DNR; 197; 38.10%), tetra- (TTR; 10; 1.93%), penta- (PNRs; 1; 0.19%), and hexa- (HNR; 2; 0.39%) nucleotide repeats (Figure 6). Among the DNR, AG/CT (74; 19.3%) is quite dominant, followed by CT/GA (50) and AT/TA (32). AAG/CTT is the most abundant unit for TNR (23.6%), followed by CCG/GGC and AAC/GTT. Interestingly, there is only one CG motif, four CCG, and 11 CGG units in *Menispermum*.

### EST-SSR Primer Design, Validation, and Cross-Species Transferability

From the RNA-seq data of *M. canadense* and *M. dauricum*, we identified 521 candidate polymorphic EST-SSRs (Supplementary Table S1). The SSR loci were then evaluated by the following criteria: zero missing rate value, 1–2 transferability value, repeat motif ≥3 bp, no primer dimmers, no hairpin structures, and no mismatches. After screening, 23 primer pairs were selected and successfully amplified, which yielded PCR products of the expected size with clear bands. Moreover, these primers were then amplified across 60 individuals from each *Menispermum* species. The number of alleles ranged from 2 to 8, with a mean of 3.70 alleles per locus. Functional annotations were characterized in the 23 developed polymorphic SSRs of *Menispermum*, and they mostly belong to general function prediction, followed by carbohydrate transport and metabolism (Supplementary Table S2).

The observed heterozygosity (*Ho*) varies from 0.059 to 0.957, and expected heterozygosity (*He*) ranges from 0.195 to 0.729 (Table 3). The polymorphism information content (PIC) varies from 0.181 to 0.69, with a mean of 0.51. Allelic richness (*R*_*S*_) across the six populations from East Asia to North America (Table 4) ranges from 1.80 (AH) to 3.41 (BJ), with the total number of detected alleles (*N*_*A*_) varied from 40 (AH) to 75 (BJ). At the regional level, the mean estimates of within-population diversity (e.g., *R*_*S*_, *He*, *Ho*) are different in United States (*R_S_* = 2.58, *He* = 0.506, *Ho* = 0.403) and China (*R_S_* = 2.40, *He* = 0.247, *Ho* = 0.281). The population BJ is found to depart from Hardy–Weinberg equilibrium significantly (*P* < 0.05; Table 4).

**TABLE 3:** Characteristics of 23 newly developed polymorphic microsatellite loci in *Menispermum*.

	**Primer sequences (5′-3′)**	**Repeat motif**	**Fluorescent dye**	**Allele size (bp)**	***T* (°C)**	***N*_*A*_**	***H*_*o*_**	***H*_*e*_**	PIC*	**GenBank accession no.**
CPSSR_1	F: GGTTCTTGAAACGGGCTTGT R: CCGTTGTCTCCCTGTTCCTC	(AAAAG)5	FAM	159–208	57	3	0.157	0.514	0.424	MK153148
CPSSR_30	F: GCGAGAACCACCTCCCTAAC R: AGTGCTGCAATGCTCATCCT	(AAG)7	HEX	119–161	55	6	0.542	0.689	0.641	MK153146
CPSSR_91	F: CTCGCACTTGTCCTGACCTT R: GCAACCGTTGAGACCGTTTC	(AGACGA)5	ROX	118–178	57	4	0.319	0.635	0.568	MK153147
CPSSR_95	F: TCGGTTAGTCCTCCTACGTG R: ATGCTGGAGCTGCTGAAACT	(AGC)7	TAMRA	129–165	56	7	0.922	0.6	0.524	MK153142
CPSSR_157	F: CGCTTTCCGTCACCAGTAGT R: TCTTCACCGATCCTCGCAAG	(CAA)6	HEX	141–183	57	2	0.383	0.493	0.371	MK153138
CPSSR_167	F: AACCACTTCACCACTGCCAT R: TGTTGGGCTAGGAGTTGCTG	(CAG)5	ROX	164–194	55	3	0.369	0.593	0.502	MK153145
CPSSR_172	F: CCACTCCGGTGAAGGAAGAG R: AGTCACCTTTGCCGGATCTG	(CAGCT)5	TAMRA	129–177	55	8	0.947	0.729	0.69	MK153140
CPSSR_175	F: TGGCATGGTGTGGAAGGATC R: ACATGCACTGTGGAGGGAAG	(CAT)6	FAM	138–174	57	5	0.957	0.62	0.553	MK153155
CPSSR_177	F: AAGACGGGCCTCATCAATCG R: TCTGCCAGGTTTTCTGCACT	(CAT)8	HEX	128–176	57	6	0.283	0.685	0.624*	MK153158
CPSSR_183	F: CGTTTGATGACGCCGTTTGT R: TCTGAGGGAGGAGGTGACTG	(CCA)5	ROX	105–135	57	5	0.226	0.516	0.474*	MK153150
CPSSR_194	F: AGAACTTGCTCTCGCTGGTC R: ACACGGCGAAAGATGTCGAT	(CCG)7	TAMRA	111–153	57	5	0.217	0.595	0.507	MK153149
CPSSR_201	F: CATCCCTTGTCTGCTTCCGT R: TCAATTTCCCTGGCGGAGTC	(CGC)7	FAM	133–175	55	8	0.567	0.634	0.597	MK153139
CPSSR_231	F: GGCCATTGCTGCAGTGTTAC R: TTCCAGAGAAATGCCGGCTT	(CTA)6	FAM	156–192	57	4	0.242	0.659	0.582*	MK153143
CPSSR_261	F: GGAGAAGCTCTATCCTCTCCCT R: CTGCGGCTCGGACAAAGTAT	(CTTCT)7	TAMRA	94–165	57	4	0.25	0.635	0.56*	MK153157
CPSSR_314	F: GTTCCAAATTGGGCCTCTGC R: CCACTGAGCCTCTCTCTTGC	(GAT)6	FAM	133–169	57	7	0.123	0.645	0.584*	MK153144
CPSSR_365	F: ACGAAGAGAAAACCGTCGCT R: CCCTTAAGCCCTACCCTCCT	(GTG)5	ROX	113–144	56	3	0.368	0.314	0.266	MK153152
CPSSR_396	F: CGACGATCTCTGCCTCGAAT R: AGGGTGCTAGGGACTCGAAT	(TAC)6	FAM	154–190	57	5	0.195	0.591	0.503*	MK153137
CPSSR_459	F: ACAGGGATTTCACGGCTGAG R: GCCTCCCTTGAACCCTTCTC	(TCG)6	HEX	109–145	56	4	0.179	0.195	0.181	MK153154
CPSSR_475	F: TCCATCACTCGTCTCCTCCT R: GATGCCCTAGACGAGAAGCC	(TCT)6	ROX	131–167	57	4	0.265	0.598	0.514	MK153159
CPSSR_502	F: TCGGTGCTGTCTCTTCTTCC R: TCAGCTTGTCTTCCCCGTTC	(TTA)6	TAMRA	162–198	57	3	0.059	0.559	0.469*	MK153151
CPSSR_517	F: GAGACGAAAGGGCAGAGTCC R: GACAGTGGCAGGAAAGTTGC	(TTC)7	FAM	126–168	56	6	0.383	0.71	0.659* 0.49	MK153153

Locus name, forward (F) and reverse (R) primer sequences, SSR motifs, size of the original fragment (bp), optimal annealing temperatures (T), the total number of alleles per locus (*NA*), observed heterozygosity (*Ho*), expected heterozygosity (*He*), polymorphism information content (PIC), and GenBank accession numbers are given. *Significantly departures from Hardy–Weinberg equilibrium (*P* < 0.05).

**TABLE 4:** Genetic parameters of the 23 microsatellite loci within six populations of *Menispermum*.^a^

**Population code (number of individuals)**	***N*_*A*_**	***Rs***	***H*_*o*_**	***H*_*e*_**	*F*_IS*_
United States – *M. canadense* (60)					
AR (20)	58	2.62	0.513	0.377	−0.243
IA (20)	61	2.82	0.492	0.388	−0.130
WI (20)	50	2.29	0.513	0.444	−0.338
Regional mean	56.33	2.58	0.506	0.403	−0.237
China – *M. dauricum* (60)					
BJ (20)	75	3.41	0.288	0.450	0.381*
AH (20)	40	1.80	0.208	0.165	−0.239
ZJ (20)	43	1.99	0.245	0.227	−0.052
Regional mean	52.67	2.40	0.247	0.281	−0.146
Total mean	54.50	2.49	0.377	0.342	−0.198

NA, total number of detected alleles; *RS*, allelic richness; *Ho*, observed heterozygosity; *He*, expected heterozygosity; *F*_*IS*_, inbreeding coefficient. ^*a*^Locality and voucher information are available in Supplementary Table S2. *Significant departures from Hardy–Weinberg equilibrium (*P* < 0.05).

In the current study, 23 candidate polymorphic EST-SSRs were developed to assess the cross-species transferability in three species *Cocculus orbiculatus*, *Sinomenium acutum*, *Stephania tetrandra* from the Menispermaceae. The transferability ratios ranged from 85 to 90%.

## Discussion

In the current study, using RNA-seq technology on the Illumina HiSeq 2500 platform, we have characterized the transcriptomes of both *Menispermum* species, *M. canadense*, and *M. dauricum*. In spite of their medicinal value (Liu et al., 2009), there is a deficiency in genetic knowledge of the genus *Menispermum*, and there are no available SSR markers.

The mean length and N50 of all unigenes of *M. canadense* were 920 bp and 1,519 bp, and those of *M. dauricum* were 945 bp and 1,528 bp, respectively. In comparison to previous studies, the mean length and N50 of all unigenes in *Menispermum* are similar to those in *Curcuma longa* (910 bp, 1,515 bp; Annadurai et al., 2013) but much higher than those in *Ipomoea batatas* (581 bp, 765 bp; Wang et al., 2010), *Cicer arietinum* (523 bp, 900 bp; Garg et al., 2011), and *Sesamum indicum* (629 bp, 947 bp; Wei et al., 2011). Additionally, the mean length is slightly lower than in *Zantedeschia rehmannii* (1,038 bp, 1,476 bp; Wei et al., 2016) and *Euphorbia fischeriana* (1,066 bp, 1,500 bp; Barrero et al., 2011). Previous studies suggest that longer mean lengths of the unigenes and larger N50 values signify accurate and effective transcriptome assembly (Chen et al., 2015; Li et al., 2018). The longer unigenes with high sequencing depth in *Menispermum* will be valuable for exploring the gene functions and the molecular mechanisms.

The unigene annotation rate was much higher in *M. dauricum* (producing 55,211 out of 78,921 significant hits) than in *M. canadense* (37,527 out of 53,712), with percentages of 69.96 and 69.87, respectively (Table 2). Based on Nr function annotation, the ratio of unigene similarity for *M. canadense*, 53.49% of annotated unigenes, exhibited similarity to *Nelumbo nucifera* (Indian lotus), which is higher than *M. dauricum*, with 42.3% (Figure 2). The limited whole chloroplast genome sequences and transcriptome sequences within public databases for *Menispermum* could influence the annotation efficiency. The gene function database (GO) predicts the physiological role of the unigenes (Kumar et al., 2014). In *M. canadense*, GO classified into 54 subcategories of three main categories at ratios of 23, 17, and 14, while in *M. dauricum*, it divided into 57 subcategories, with 23, 19, and 15, respectively, in the three main categories (Figure 3). This result is also comparable with prior studies on *C. alismatifolia* (Taheri et al., 2019), in which unigenes were grouped into 51 subcategories within three categories at 23, 17, and 11, and *Rhododendron rex* (Zhang et al., 2017), with 62 subcategories at 25, 20, and 17. In addition, several unigenes identified in the annotated GO database are responsible for cold shock (K09250), heat tolerance (K09419), and osmotic stress (GO:0009266) in *Menispermum* (Mutlu et al., 2013; Zhang et al., 2017). These results illustrate the involvement of diverse molecular functional unigenes in varied metabolic pathways.

Of all KEGG pathways, global and overview maps were dominant, followed by carbohydrate metabolism, translation, folding, sorting and degradation, in *M. canadense*, while in *M. dauricum*, global and overview maps were dominant, followed by translation, carbohydrate metabolism, and lipid metabolism. Moreover, 135 pathways mapped against the KEGG database in *M. canadense*, which is higher than for *M. dauricum*, which significantly matched with only 128 pathways. Our findings generated rich genetic and genomic information that will facilitate further research in biochemistry, gene function discovery, physiological genetics, molecular genetics, and the biological pathways in *Menispermum* or related species.

Microsatellites have been extensively used in forensics, phylogeography, genomic mapping, population genetics, conservation genetics, molecular breeding, and determining parentage over the past several decades (Ellegren, 2000; Esselink et al., 2004). The frequency and distribution of microsatellites are dependent on an array of factors such as the size of the dataset, tools, and criteria used for mining. EST-SSRs are from the transcribed DNA regions and exhibit major advantages for their presence in the functional genes, consistent amplification efficiency, and high cross-species transferability rates (Scott et al., 2000; Gupta et al., 2003). To our knowledge, no SSR markers are available in *Menispermum*. The current study presents the first mining and development of EST-SSRs in *Menispermum*. To date, several tools have been used for the assessment of SSR and polymorphism, such as MISA (Thiel, 2003), SSRLocator (Da Maia et al., 2008), and GMATA (Wang and Wang, 2016). However, they all have some deficiencies, such as long running time, poor capacity for a large dataset, a need to manually screen for polySSR, or inability to dealing with large genomes or multiple individuals. CandiSSR (Xia et al., 2016) enables users to find the putative polymorphic SSR from several species with great ease and efficiency from the genomes and also from the transcriptomes. In the current study, we used CandiSSR for the development of putative polymorphic EST-SSR markers. From the transcriptome of the two species of *Menispermum*, 521 polymorphic EST-SSR markers were proficiently mined. The most dominant nucleotide repeat motifs are AG/CT (19.3%) and AAG/CTT (23.6%) for di- and trinucleotide repeat motifs, which are similar to *Heveabra siliensis* (rubber tree) (Li et al., 2012) and *Sesamum indicum L.* (sesame) (Zhang et al., 2012). Trinucleotides are the most abundant repeat motifs in *Menispermum* because, in open reading frames (ORFs), there may be no hindrance from the insertions, deletions, or any mutations within the translated regions, whereas frameshift mutation may restrict the development of other types of SSR motifs (Metzgar et al., 2000; Chen et al., 2015). Our results robustly support and expand the belief that there is a low GC content in eudicots, because only one CG, four CCG, and eleven CGG motifs were found. The rarity of GC/CG, CCG/CGG repeat units (Kumpatla and Mukhopadhyay, 2005; Chen et al., 2015) has been reported in plenty of dicotyledonous plants, for example, *Medicago truncatula* (Eujayl et al., 2004), *Raphanus sativus* (Wang et al., 2013), and *Ipomoea batatas* (Wang et al., 2011). Previous studies on dicotyledonous plants (Morgante et al., 2002; Kumpatla and Mukhopadhyay, 2005) also illustrated that the tri-nucleotide AAG motif may be significantly prominent, such as in *Cucumis sativus* (Hu et al., 2010), *Ricinus communis* (Tan et al., 2014), and *Sesamum indicum* (Wei et al., 2011).

In the present study, 23 polymorphic EST-SSR markers were selected to evaluate the genetic diversity among *Menispermum* populations. In the developed polymorphic EST-SSRs, the number of alleles ranges from 2 to 8 with a mean of 4.86, *Ho* = 0.38, *He* = 0.58, and PIC = 0.49 (Table 3), which depict a high level of polymorphism in *Menispermum*. Besides, *M. canadense* demonstrated higher genetic diversity, with *N_A_* = 56.33 and *R_S_* = 2.58, than *M. dauricum*, with *N_A_* = 4.50 and *R_S_* = 2.49 (Table 4). The genetic diversity patterns found here may help in our future study on the East Asia-North America disjunctive distribution of *Menispermum*.

The 23 polymorphic EST-SSR markers were also tested for transferability in three other Menispermaceae species. The primers were successfully amplified with multiple bands in all the species except for SR1. The transferability ratio was 85–90%, which is comparable or higher than that obtained in *Festuca arundinacea* (92%; Saha et al., 2004), *Medicago truncatula* (89%; Eujayl et al., 2004), *Epimedium sagittatum* (85.7%; Zeng et al., 2010), and *Cumumis melo* (12.7%; Fernandez-Silva et al., 2008). EST-SSRs depict a higher transferability ratio owing to the high conservation of genetic markers in comparison to gSSRs, which are derived through genomic libraries. Hence, the high transferability across the family Menispermaceae presents valuable information resources for the development of molecular markers and evolutionary studies. Furthermore, the novel polymorphic EST-SSR markers and the characterization of *Menispermum* transcriptomes will offer a comprehensive source of specific genes along with in-depth knowledge of their pathways.

## Materials and Methods

### RNA Isolation, Construction of cDNA Library, Illumina Sequencing and Reads Filtering

Fresh leaves from *M. canadense* and *M. dauricum* were sampled, cleaned, and placed in liquid nitrogen until RNA extraction. RNA was isolated by using an RNeasy Plant kit (Qiagen Bioinformatics, Germany), and quality and quantity were evaluated with an Agilent 2100 Bioanalyzer (Agilent Technologies, United States) and NanoDrop^TM^ (Agilent RNA 6000 Nano Kit) to determine the purity of the RNA samples. Preparation of two cDNA libraries was then performed using a NEBNext Ultra TM RNA-seq Library Preparation Kit (New England Bio Labs, United States). Oligo-(dT) magnetic beads were used to isolate poly-A from the total RNA. cDNA fragments were purified by MinElute Kit (Qiagen Bioinformatics, Germany) and resolved with the EB buffer for the reparation of the end with the addition of single nucleotide A (Adenine). The first-strand cDNA was synthesized by random hexamer primers and superscript III reverse transcriptase. After the second-strand cDNA preparation, end-repaired and dA-tailed fragments connected with the sequencing adapters. Adapter ligated cDNA libraries (about 500 bp) were amplified and sequenced through the Illumina HiSeq 2500 platform (Illumina Inc., BGI, Shenzhen, China). Raw data (2 × 150 bp paired-end reads) filtration was done by FASTX-TOOLKIT (Gordon and Hannon, 2010) version 0.0.14, which removed the reads with adapters with more than 5% unknown bases (N) and low-quality reads with quality less than 15% and greater than 20%. After filtering, the remaining reads were clean reads.

### *De novo* Assembly and Annotation

TRINITY (Grabherr et al., 2011) version 2.0.6 was employed to perform the *de novo* assembly with clean reads. Paired-end reads were mapped to contigs from the same transcripts and distances. The resulting sequences with the lowest number of Ns were generated as unigenes. To avoid redundancy, TGICL (Pertea et al., 2003) version 2.0.6 (-l 40 -c 10 -v 25 -O ’-repeat_stringency 0.95 - minmatch 35 -minscore 35’) was then used to cluster 86,883 and 104,064 transcripts into 53,712 and 78,921 non-redundant unigenes in *M. canadense* and *M. dauricum*, respectively. The raw sequencing data from both of the species were deposited in the Sequence Read Archive (SRA) of NCBI (National Center for Biotechnology Information and their accession numbers were acquired. BLASTN version 2.2.23 with default parameters^1^ and BLASTX (e-value <10^–5^) were used to align Unigenes to the Nt, Nr^2^, KOG^3^, KEGG^4^, and Swiss-Prot^5^ databases to perform the annotations, while Blast2GO version2.5.0^6^ was used for Nr annotation to perform the GO annotation.

### EST-SSR Marker Development, Mining, and Primer Design

Through CandiSSR (Xia et al., 2016), we successfully mined 521 candidate polymorphic EST-SSRs with a 20-bp mean length (Supplementary Table S1) by comparing the unigenes of two *Menispermum* species. In CandiSSR, the parameters were set to sequence length 100, blast e-value 1e^–10^ cutoff, 95 cutoff blast identity, and 95 cutoff blast coverage. Based on the Primer3 package (Koressaar and Remm, 2007), primers were generated automatically for each SSR locus. Optimal polySSR primers were selected under three criteria: (1) missing rate value = zero, (2) transferability value = 1; (3) repeat motif ≥3 bp. Primer dimers, hairpin structures, or mismatches were checked in OLIGO (Rychlik, 2007) version 7 (Molecular Biology Insights Inc., Colorado Springs, CO, United States). With this strict screening strategy, we sorted out 23 primer pairs for amplification. Electrophoresis peaks were assessed through GeneMarker (Holland and Parson, 2011) version 2.4.0. In total, 23 primer pairs that had high variations and stable repetition were selected for further analysis. The primer sequences analyzed in this study were submitted to GenBank (Table 3).

### EST-SSR Amplification and Transferability in Cross-Species

To perform PCR, a forward primer with an M13 sequence (5′−TGTAAAACGACGGCCAGT−3′) at the 5′ end was synthesized for all loci, and four fluorescents (FAM, ROX, HEX, and TAMRA) were labeled with a universal M13 primer (5′−TGTAAAACGACGGCCAGT−3′). The two currently recognized *Menispermum* species were selected for population genetic studies. Within the genus, three populations from *M. canadense* ranging from North America to Canada and three from *M. dauricum* endemic to China were selected. Three populations with 20 individuals from each species (Supplementary Table S3) were tested for population genetics analyses. DNA was extracted from silica gel-dried leaves through Plant DNAzol (Invitrogen Life Technologies). The DNA quality was assessed through electrophoresis on the gel with 0.8% agarose colored with 1% GelRed (Biotium) with the reference of a 1,000-bp marker (TaKaRa, Dalian, Liaoning, China), on the basis of the integrity and intensity of the band on the gel.

PCR amplifications were performed through the two−step PCR protocol (Schuelke, 2000) by following the Tsingke PCR kit protocol (Tsingke Biotech Company, Beijing, China). In the first step, a final volume of 25 μL PCR mixture was obtained containing 1.0 μL DNA template, 12.5 μL Mix AmpliTaqGold 360 (Thermofisher Biotech Company, Applied Biosystems, Foster City, CA, United States), 9.5 μL of distilled water, and 1.0 μL of forward primer (which was produced with an 18-bp M13 tail 5′-TGTAAAACGACGGCCAGT-3′ at the 5′ end) and 1.0 μL reverse primers. The PCR amplification procedure was initial denaturation for 5 min at 95°C, 35 cycles of 30 s at 95°C, 45 s at annealing temperature (optimal primer temperature TM), 30 s syntheses at 72°C, and finally a 10-min extension period at 72°C with a 4°C holding temperature. In the second step, a 30-μL final volume was obtained containing 3 μL of the first PCR products, 1 μL fluorescent-labeled (FAM, ROX, HEX, TAMRA) universal M13 primer and 1 μL of reverse primer, 15 μL PCR Mix, and 10 μL distilled water. SSR loci were amplified under the following conditions: 2 min at 94°C; 35 cycles of 94°C for 60 s, 30 min at 57°C, 60 s at 72°C, and finally a 10-min extension step at 72°C and a 4°C holding temperature. Electrophoresis peaks were assessed through Gene Marker version 2.4.0 (Soft Genetics, State College, PA, United States). A total of 23 primer pairs demonstrating stable repeatability with high variations were picked for further analysis.

Cross-species transferability was tested among three Menispermaceae species, *Cocculus orbiculatus*, *Sinomenium acutum*, and *Stephania tetrandra*. Five individuals from each were assessed using the same DNA extraction and PCR procedure. A gene marker was used to assess the peaks.

### Population Genetic Diversity Analysis

Overall genetic parameters, i.e., the number of alleles, observed and expected heterozygosities, and PIC (polymorphism information content) were calculated for the assessment of genetic polymorphism per locus using CERVUS (Kalinowski et al., 2007) version 3.0.3 and GenAlEx (Peakall and Smouse, 2006) version 6.5. The significance of deviations from Hardy–Weinberg equilibrium, given by *F*_*IS*_ deviation, were tested by FSTAT (Goudet, 2001) version 2.9.3.

## Data Availability Statement

The datasets generated for this study can be found in the *M. canadense*: SRP166813, *M. dauricum*: SRP166814, SSR loci: MK153148, MK153146, MK153147, MK153142, MK153138, MK153145, MK153140, MK153155, MK153158, MK153150, MK153149, MK153139, MK153143, MK153157, MK153144, MK153152, MK153137, MK153154, MK153159, MK153151, MK153153.

## Author Contributions

CF, PL, and FH made substantial contributions to the conception and design of this research. In particular, PL and SW collected the materials. FH performed the experiments, wrote the manuscript, organized the contents of the article, and prepared figures. FH and GY analyzed the data. PL, SW, and CF revised the manuscript.

## Conflict of Interest

The authors declare that the research was conducted in the absence of any commercial or financial relationships that could be construed as a potential conflict of interest.

## Footnotes

1 http://blast.ncbi.nlm.nih.gov/Blast.cgi

2 ftp://ftp.ncbi.nlm.nih.gov/blast/db

3 http://www.ncbi.nlm.nih.gov/KOG

4 http://www.genome.jp/keg

5 http://ftp.ebi.ac.uk/pub/databases/swissprot

6 http://www.blast2go.com

## Supplementary Material

FIGURE S1 The length distribution of the transcripts in *Menispermum canadense* (MC) and *M. dauricum* (MD). The x-axis represents the lengths of all the trinity sequences, and the y-axis represents the numbers of trinity sequences with certain length.

TABLE S1 Designed EST-SSR from CandiSSR.

TABLE S2 EST-SSR functional annotation in *Menispermum*.

TABLE S3 Locality and voucher information for *Menispermum* used in this study. Voucher specimens are deposited at the herbarium of Zhejiang University (HZU), Hangzhou, Zhejiang, China.
