# Supplementary figures and images for: De novo Transcriptome Assembly, Gene Annotation and SSR Marker Development in the Moon Seed Genus Menispermum (Menispermaceae)
Source: Front Genet. 2020 May 8;11:380. doi: 10.3389/fgene.2020.00380 (PMC7227793; doi:10.3389/fgene.2020.00380)

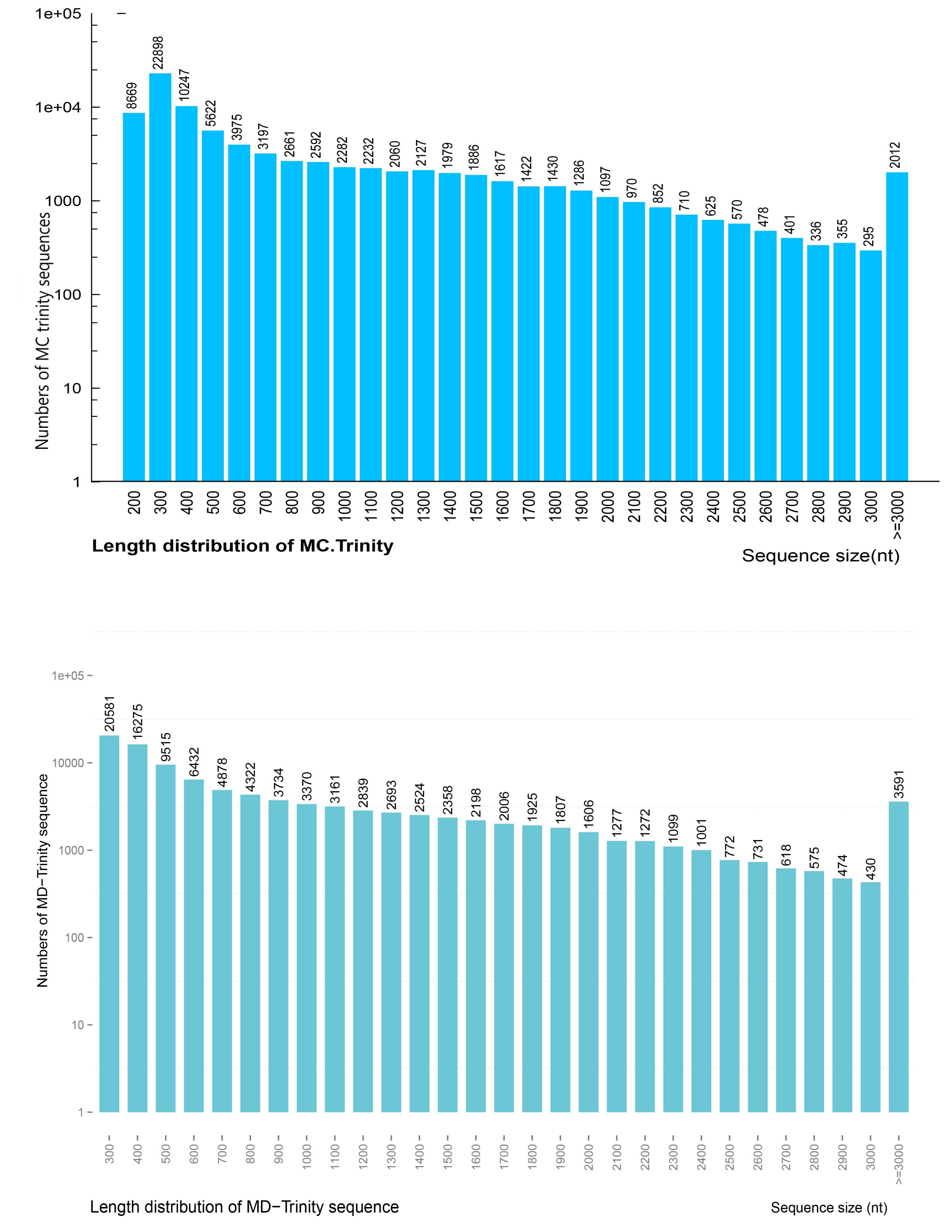

Supplement: FIGURE S1 — The length distribution of the transcripts in Menispermum canadense (MC) and M. dauricum (MD). The x-axis represents the lengths of all the trinity sequences, and the y-axis represents the numbers of trinity sequences with certain length. [file Image_1.JPEG]
