# Supplementary material for: De novo Transcriptome Assembly, Gene Annotation and SSR Marker Development in the Moon Seed Genus Menispermum (Menispermaceae)
Source: Front Genet. 2020 May 8;11:380. doi: 10.3389/fgene.2020.00380 (PMC7227793; doi:10.3389/fgene.2020.00380)
Supplement: TABLE S3 — Locality and voucher information for Menispermum used in this study. Voucher specimens are deposited at the herbarium of Zhejiang University (HZU), Hangzhou, Zhejiang, China. [file Table_3.DOCX]

| Species | Population code | Voucher number | Locality | Geographic coordinates | Altitude (m) |
| --- | --- | --- | --- | --- | --- |
| *M. canadense* | AR | *P. Li LP162210* | Mena, AR, USA | N34°35'44", W94°14'28" | 384 |
|  | IA | *P. Li LP162290* | Des Moines, IA, USA | N41°32'56", W93°42'38" | 288 |
|  | WI | *P. Li LP162439* | Madison, WI, USA | N43°02'52", W89°25'21" | 260 |
| *M. dauricum* | BJ | *P. Li LP161648* | Mentougou, Beijing, China | N39°57'47", E115°25'50" | 1186 |
|  | AH | *P. Li LP185080* | Tongling, Anhui, China | N30°49'11", E117°52'23" | 373 |
|  | ZJ | *P. Li LP185440* | Lin’an, Zhejiang, China | N30°09'34", E119°06'24" | 151 |

**Table S3.** Locality and voucher information for *Menispermum* used in this study.Voucher specimens are deposited at the herbarium of Zhejiang University (HZU), Hangzhou, Zhejiang, China.
